# Predictive Value of End-Tidal CO_2_, Arterial CO_2_, CO_2_ Gap and Ratio for 30-Day Mortality in Emergency Department Intubations: A Prospective Analysis

**DOI:** 10.3390/medicina62061075

**Published:** 2026-06-01

**Authors:** Melis Efeoglu Sacak, Emir Unal, Ozgecan Gorman, Aykut Ozkan, Nurseli Bayram, Ozge Onur, Haldun Akoglu, Arzu Denizbasi Altinok

**Affiliations:** 1 Department of Emergency Medicine, Marmara University School of Medicine, 34899 Istanbul, Turkey; emirunal@gmail.com (E.U.); ozberkozge@gmail.com (O.O.); drhaldun@gmail.com (H.A.); denizbasi@gmail.com (A.D.A.); 2 Department of Emergency Medicine, King’s College Hospital NHS Foundation Trust, London SE5 9RS, UK; ozgeegzo@gmail.com; 3 Department of Emergency Medicine, Marmara University Pendik Training and Research Hospital, 34899 Istanbul, Turkey; md.aykutozkan@gmail.com (A.O.); nurselibyrm@gmail.com (N.B.)

**Keywords:** end-tidal CO_2_, PaCO_2_–ETCO_2_ difference, intubation, capnography, respiratory failure

## Abstract

*Background and Objectives*: To evaluate the prognostic value of end-tidal carbon dioxide (ETCO_2_), arterial carbon dioxide (PaCO_2_), their difference (ΔCO_2_ = PaCO_2_–ETCO_2_), and the ETCO_2_/PaCO_2_ ratio for 30-day mortality in adult patients intubated for acute respiratory failure in the emergency department (ED). *Materials and Methods*: In this single-center, prospective observational study, we enrolled consecutive adults intubated in the ED who had at least two paired arterial blood gas and capnography measurements within 90 min post-intubation. PaCO_2_ and ETCO_2_ were recorded at 0, 30, 60, and 90 min, and ΔCO_2_ and ETCO_2_/PaCO_2_ were calculated at each time point. Between-group comparisons, repeated-measures analyses, and logistic regression were used to explore associations, and prognostic performance was assessed using receiver operating characteristic curves. *Results*: Among 100 patients (36% female); 30- and 90-day mortality were 64% and 70%, respectively. Non-survivors were older, had lower mean arterial pressure after intubation, higher inflammatory and coagulation markers, and more frequent pneumonia and pulmonary embolism. ETCO_2_ values were consistently lower in non-survivors at 0, 30, and 60 min, while PaCO_2_ was also lower at early time points. ΔCO_2_ and the ETCO_2_/PaCO_2_ ratio did not differ between survivors and non-survivors. Among all CO_2_ metrics and time points, only ETCO_2_ showed low-to-moderate discrimination for 30-day mortality (area under the curve 0.65–0.70). *Conclusions*: In ED patients emergently intubated for acute respiratory failure, absolute ETCO_2_ values in the early post-intubation period provide modest prognostic information for short-term mortality, whereas ΔCO_2_ and ETCO_2_/PaCO_2_ do not appear to add prognostic value beyond ETCO_2_ alone.

## 1. Introduction 

End-tidal carbon dioxide (ETCO_2_) monitoring via capnography has become a cornerstone of modern emergency and critical care. Persistently low ETCO_2_ values during cardiopulmonary resuscitation correlate with inadequate cardiac output, poor perfusion, and poor prognosis [1,2,3,4]. Similarly, in pulmonary embolism (PE), where ventilation–perfusion mismatch is prominent, ETCO_2_ levels fall despite elevated or normal PaCO_2_, offering diagnostic and prognostic insight [5,6,7,8]. Therefore, several metrics, such as the difference between arterial carbon dioxide pressure and ETCO_2_ (PaCO_2_–ETCO_2_ difference, or “CO2 gap”), and the ratio of ETCO_2_ to PaCO_2_ have gained attention. In healthy individuals, this difference remains narrow—generally 2–5 mmHg—because alveolar CO_2_ closely approximates arterial CO_2_ [2]. However, in critically ill states, the gap widens due to increased physiologic dead space, severe V/Q mismatch, and low cardiac output [9,10,11,12]. This widening not only signals impaired gas exchange but also provides an indirect measure of circulatory efficiency. For example, Shetty et al. demonstrated that a widened CO_2_ gap in emergency department patients with suspected sepsis was independently associated with adverse outcomes [13]. Kim et al. reported that an increased PaCO_2_–ETCO_2_ difference predicted in-hospital mortality in post–cardiac arrest patients [14]. In sepsis and septic shock, a CO_2_ gap > 6 mmHg has been associated with higher mortality [13], while in post-arrest populations, differences > 10 mmHg were linked to poor outcomes [14].

Studies have shown that a widened gap not only supports diagnosis but also predicts outcomes in patients with PE [15,16,17,18,19], and the PaCO_2_–ETCO_2_ difference has been correlated with severity and prognosis in COPD exacerbations and asthma [6,7,8]. Thus, the CO_2_ gap has emerged as a potentially powerful, non-invasive biomarker that integrates information from both ventilatory and circulatory domains.

Despite this growing body of evidence, studies investigating the prognostic role of delta CO_2_ or PETCO/PCO ratio in patients intubated due to acute respiratory failure are lacking. This group represents a unique population: they are physiologically unstable, often exhibit complex interactions between pulmonary and circulatory pathophysiology, and face high short-term mortality. Identifying reliable, noninvasive prognostic indicators in this context could provide clinicians with rapid, bedside tools to stratify risk, tailor interventions, and improve outcomes.

Therefore, in this study, we aimed to prospectively evaluate the predictive value of ETCO_2_, PaCO_2_ and ΔCO_2_, and the PETCO/PCO ratio for 30-day mortality in a high-risk population.

## 2. Methods

### 2.1. Study Design and Setting

This is a prospective, observational, prognostic, single-center study conducted at an academic emergency medicine department with an annual patient volume of 300,000. This study was approved by the local Clinical Research Ethics Committee, with protocol code 09.2020.381, and was carried out in accordance with the Declaration of Helsinki. Informed consent procedures were followed in accordance with institutional policies for emergency and critical care settings.

### 2.2. Study Population

Consecutive adult patients who were intubated in the emergency department due to acute respiratory failure were eligible for inclusion in this study. Written informed consent was obtained from all participants prior to enrollment. Inclusion criteria were the availability of at least two paired measurements of arterial blood gas (ABG) and capnography within the first 90 min post-intubation (0 and 30 min mandatory). Exclusion criteria were (i) major technical artifacts invalidating ETCO_2_ measurement (e.g., circuit leakage), (ii) patients with preexisting tracheostomy, (iii) pregnancy, and (iv) consent withdrawal.

### 2.3. Data Collection, Predictor Variables and Measurements

Demographics (sex, age, reason for intubation, diagnostic groups), vital signs (SBP, DBP, MAP, temperature, HR, and Glasgow Coma Scale), arterial blood gases, and routine biochemistry results at presentation were collected from bedside patient charts and the hospital information system by researchers. Diagnostic groups (pneumonia, COPD exacerbation, pulmonary embolism, acute cardiac conditions, etc.) were confirmed through clinical assessments, imaging studies, consultations, and discharge records.

PaCO_2_ was obtained from arterial samples collected in heparinized syringes and analyzed at the central laboratory. ETCO_2_ was continuously monitored with sidestream capnography immediately after intubation. PaCO_2_ and ETCO_2_ were measured at intubation (0 min) and then at 30, 60, and 90 min, with recordings made by the physicians in charge. The difference (ΔCO_2_ = PaCO_2_–ETCO_2_) and the proportion (PETCO/PCO = ETCO_2_/PaCO_2_) of CO_2_ measurements were calculated for each time point during data analysis.

### 2.4. Outcomes

The primary outcome was all-cause mortality within 30 days after emergency department intubation. Secondary outcomes included (i) all-cause mortality at 90 days, (ii) changes over time in PaCO_2_, ETCO_2_, ΔCO_2_, and PETCO/PCO during the first 90 min post-intubation, (iii) mortality patterns within subgroups based on underlying diagnoses (e.g., pneumonia, pulmonary embolism), and (iv) the prognostic value of a model built from available predictor variables. Mortality was verified using the national death registry system.

### 2.5. Sample Size Considerations

The target sample size was set at 100 patients to ensure adequate accuracy for agreement analysis between PaCO_2_ and ETCO_2_, as well as enough events for exploratory prognostic modeling. For Bland–Altman analysis, we assumed an a priori standard deviation of approximately 6 mmHg for the PaCO_2_–ETCO_2_ difference, a value chosen to sit at the center of the 5–8 mmHg range reported in prior bedside paired-measurement studies in critically ill and acutely dyspneic patients [9,11,20,21]. With *n* = 100, this assumption yields 95% limits of agreement with a confidence-interval half-width of approximately ±2 mmHg, which we considered clinically acceptable.

Regarding prognostic assessment, with an expected 30% 30-day mortality rate (~30 events), this sample size allows estimating an area under the ROC curve (AUC) of 0.70 with a 95% confidence interval of roughly ±0.12. It supports parsimonious logistic regression with one to two candidate prognostic variables using penalized methods. This strategy aligns with recommended principles for prognostic factor studies outlined in the PROGRESS and TRIPOD frameworks. Missing data varied across time points, with more missing data at 60 and 90 min. Analyses were conducted using a complete-case approach, with effective sample sizes provided for each test. Cross-sectional analyses (Table 1) used the full cohort (N = 100). Analyses that required paired 0 and 90 min capnographic measurements (Table 2, upper rows) used a complete-case sub-cohort of 57 patients (23 survivors, 34 non-survivors); the remaining 43 patients had missing 60 or 90 min measurements due to early ICU transfer, extubation, or death within the observation window. To verify that this complete-case constraint did not bias the temporal contrasts, we additionally re-ran the analyses on the full N = 100 using last observation carried forward (LOCF) impu tation (Table 2, lower rows).

### 2.6. Statistical Analysis

Continuous variables were summarized as medians with interquartile ranges, and categorical variables as counts with percentages. Between-group comparisons (survivors vs. non-survivors) were conducted using the Mann–Whitney U test for continuous variables and the χ^2^ or Fisher’s exact test for categorical variables, as appropriate. Agreement between PaCO_2_ and ETCO_2_ was assessed with Bland–Altman analysis, reporting bias, limits of agreement, and 95% confidence intervals; proportional bias was evaluated by linear regression of the differences on the means.

Temporal changes in PaCO_2_, ETCO_2_, ΔCO_2_, and PETCO/PCO across serial measurements (0, 30, 60, and 90 min post-intubation) were analyzed using repeated-measures ANOVA with Greenhouse–Geisser correction, including tests for interaction between time and survival status. We chose this combined strategy deliberately: cross-sectional between-group contrasts of skewed CO_2_ distributions at each time point were performed with the non-parametric Mann–Whitney U test, whereas the within-subject temporal evolution and its interaction with survival status were tested with repeated-measures ANOVA, which targets a fundamentally different (interaction) hypothesis and is robust at our sample size under Greenhouse–Geisser correction (Oberfeld & Franke 2013) [22]. As a sensitivity analysis, we re-ran each temporal model as a linear mixed-effects model with a random intercept per patient, time as a within-subject factor, and the time × survival interaction as the fixed effect of interest; results were qualitatively identical.

Logistic regression was used to derive models fit for predicting 30-day mortality, with results expressed as odds ratios and 95% confidence intervals. Restricted models were used to account for the limited number of events, and penalized likelihood methods (Firth’s correction) were applied where appropriate to reduce small-sample bias. Discriminative performance was evaluated using receiver operating characteristic (ROC) curve analysis and the area under the curve (AUC), along with 95% confidence intervals. Calibration was explored graphically and with the Hosmer–Lemeshow test. All tests were two-sided, and a *p*-value < 0.05 was considered statistically significant. Analyses were performed using the Jamovi statistical package based on R, version 2.7.6 (Jamovi project).

## 3. Results

Of 100 patients included in the study, 36 (36%) were women. The mortality rate was 64% for one month and 70% for three months. Demographics, baseline laboratory parameters, presenting vital signs, and contributing diagnoses are listed in Table 1.

Contributing diagnoses were not mutually exclusive; the most frequent was pneumonia (64.0%), followed by COPD exacerbation (40.0%) and congestive heart failure (39.0%). Twenty-nine patients (29.0%) carried more than one contributing diagnosis (e.g., pneumonia superimposed on COPD or on chronic heart failure). The study was a priori designed as a heterogeneous, all-comers prognostic-factor cohort, reflecting the real-world ED intubation case mix.

Higher age, lower mean arterial pressure after intubation, and elevated inflammatory (CRP and PCT) and coagulation markers (INR) were associated with 30-day mortality in univariate analysis. Pneumonia and pulmonary embolism were also strongly associated with 30-day mortality.

Across all time points, ETCO_2_ values were significantly lower in non-survivors, particularly at 0, 30, and 60 min (*p* ≤ 0.01). PaCO_2_ was also lower at baseline and 30 min. In contrast, ΔCO_2_ did not distinguish survivors from non-survivors at any time point (all *p* ≥ 0.217) (Table 2).

**Figure 1:**
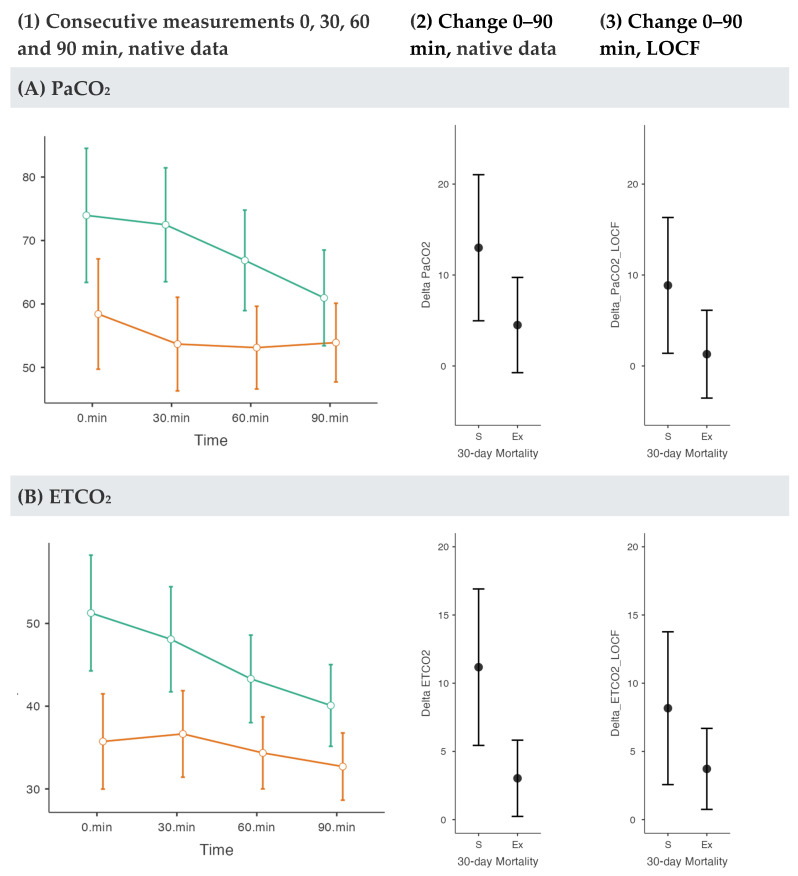

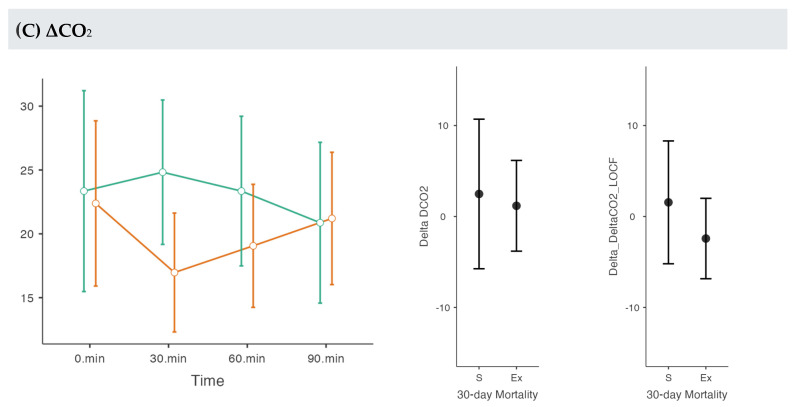
Error plots of consecutive measurements in 30-day mortality groups (green = survivors, red = non-survivors). (**A**) PaCO2, (**B**) ETCO2, (**C**) ΔCO_2_. (1) Consecutive measurements, (2) change in 0–90 min, native data, (3) change in 0–90 min, LOCF. Whiskers represent 95% CI. LOCF: last observation carried forward, PaCO_2_: partial pressure of carbon dioxide, ETCO_2_: end-tidal carbon dioxide, ΔCO_2_: PaCO_2_–ETCO_2_.

There was no statistically significant interaction effect between time and 30-day mortality in repeated-measures ANOVA. Patients with 30-day survival presented with significantly higher levels of CO2 parameters, and this trend continued after the intubation. We observed a rapid decline and supra-normalization in PaCO2 and ETCO2 levels of patients in the survivor group after intubation. However, PaCO_2_ and ETCO_2_ levels remained relatively stable in the non-survivor group (Figure 1).

The mean change in 90 min was not statistically significant for PaCO_2_ and ΔCO_2_ but significantly higher in the survivor group for ETCO_2_ (Figure 1 and Table 2). This significance was lost when missing data was replaced with the last observation carried forward method (Table 2).

The 90-day follow-up analysis findings were similar to the 30-day findings: ETCO_2_ was consistently lower in non-survivors, with the strongest differences at 0 and 60 min (both *p* < 0.001). PaCO_2_ was variably different, while ΔCO_2_ again showed no prognostic value.

Among all CO_2_ parameters (PCO2, ETCO, COGap, ETCO/PCO) and different measurement points (0 to 90 min), only ETCO2 showed a statistically significant, low-to-moderate predictive utility for 30-day mortality. The AUROC for ETCO_2_ and 30-day mortality was significant at all time points between 0.65 and 0.70. The clinical utility ranges for sensitivity, specificity, positive and negative likelihood ratios were 45–85%, 43–86%, 1.5–3.6 and 0.33–0.68, respectively, at thresholds determined by the Youden J index.

The 6-percentage-point increment in all-cause mortality between days 30 and 90 (from 64% to 70%, i.e., six additional deaths) was not uniformly distributed across diagnostic subgroups. Of these six late deaths, three occurred in patients with lung cancer, two in patients whose primary contributing diagnosis was pneumonia, and one in a patient with COPD; no additional deaths occurred between days 30 and 90 in patients whose primary contributing diagnosis was congestive heart failure or pulmonary embolism (the latter because all eight PE patients had already died within 30 days). The clustering of late deaths in patients with underlying malignancy is biologically consistent with the natural trajectory of advanced lung cancer, in which post-acute deaths typically reflect tumor progression rather than the index respiratory event.

## 4. Discussion

In this prospective, observational study, we evaluated the prognostic value of arterial and end-tidal CO_2_ parameters in adult patients intubated for acute respiratory failure in the emergency department. The principal finding is that ETCO_2_ and PaCO_2_ measured at multiple time points (0, 30, 60, and 90 min post-intubation) were significantly lower in non-survivors compared to survivors, whereas ΔCO_2_ (the PaCO_2_–ETCO_2_ difference) showed no significant association with 30-day mortality at any time point. These findings suggest that in ED patients requiring emergent intubation for acute respiratory failure, absolute values of ETCO_2_ and PaCO_2_ may be more prognostically informative than their difference.

The existing literature presents a heterogeneous picture regarding the prognostic value of the arterial–end-tidal CO_2_ gradient across different clinical contexts. Several studies have reported significant associations between widened CO_2_ gaps and adverse outcomes. Prior studies in sepsis and post–cardiac arrest cohorts have linked a widened CO_2_ gap and low ETCO_2_ to higher mortality [13,14]. In sepsis and emergency risk stratification, Shetty et al. demonstrated in a prospective ED study of 412 patients with dyspnea that CO_2_ gap ≥ 10 mmHg achieved 100% sensitivity and 70% specificity for predicting the need for ventilatory support, with an AUC of 0.95 [23]. These studies established the CO_2_ gap as a potentially powerful prognostic tool in undifferentiated ED populations. In trauma populations, the evidence for the CO_2_ gap appears particularly robust. Doppmann et al. studied 105 traumatic brain injury patients transported by helicopter and found that the mean admission CO_2_ gap was significantly larger in non-survivors (16.96 ± 9.75 mmHg) than survivors (10.65 ± 6.90 mmHg), with an adjusted odds ratio of 2.692 per 7.5 mmHg increase (95% CI 9.7–42.3 mmHg, *p* = 0.009). They found that the mean gap was no longer significantly different between non-survivors and survivors after 24 h [20]. Similarly, in a large trauma surgery series of 501 patients, Tyburski et al. reported that the mean Pa–ETCO_2_ difference was <10 mmHg in survivors and >10 mmHg in non-survivors, at all times [24]. A recent study examined a similar ED population intubated for a different indication (COVID-19). Karaali et al. prospectively studied 48 COVID-19 patients who were urgently intubated in the ED and found that survivors had higher PETCO_2_ immediately after intubation and at 15 min compared with non-survivors (*p* = 0.014 and *p* = 0.015, respectively). Importantly, this study focused on absolute ETCO_2_ values rather than the CO_2_ gap, and their findings—that lower ETCO_2_ predicts mortality—align with our observation that ETCO_2_ was significantly lower in non-survivors. However, their study was limited to a single etiology, whereas our cohort included diverse causes of acute respiratory failure [25].

Critically, the most directly comparable study to ours shows findings that closely align with ours. Upchurch et al. conducted a retrospective observational cohort study of 519 adults who were intubated and invasively mechanically ventilated in an academic ED between 2009 and 2016 [21]. This study shares notable methodological similarities with our work: it examined ED patients requiring intubation, measured early post-intubation CO_2_ parameters, and assessed in-hospital mortality as the primary outcome. The Upchurch study found that 63% of patients had an elevated PaCO_2_–PETCO_2_ gap (>5 mmHg). Yet, there was no difference in in-hospital mortality between those with elevated versus non-elevated gaps in unadjusted analysis (25% vs. 26%, *p* = 0.829). After multivariable adjustment for age, APACHE II score, and intubation indication, the adjusted odds ratio for mortality with an elevated gap was 0.81 (95% CI 0.53–1.26), indicating no significant association. This null finding in the largest published ED intubation cohort is especially noteworthy because it directly contradicts the positive associations reported in more specific populations (sepsis, ARDS, trauma, cardiac arrest) while also strongly supporting our own findings [21]. The consistency between Upchurch’s retrospective analysis of 519 patients and our prospective evaluation of 100 patients, despite different study designs and settings, suggests that the CO_2_ gap may not be a reliable prognostic marker across diverse ED intubation populations. Similarly, in COPD and acute asthma, PaCO_2_–ETCO_2_ coupling degrades with disease severity [10,11,26], offering an additional rationale for why ΔCO_2_ may underperform in mixed critical cohorts.

Beyond CO_2_ metrics, several routinely available variables differed significantly between survivors and non-survivors in our cohort: older age, lower post-intubation mean arterial pressure (MAP_0_), higher inflammatory markers (CRP, procalcitonin), and higher INR. These signals are biologically coherent. Age and post-intubation MAP capture, respectively, physiologic reserve and peri-intubation hemodynamic vulnerability—well-established determinants of short-term mortality in mechanically ventilated cohorts. Elevated CRP and procalcitonin reflect the substantial contribution of bacterial pneumonia (64% of the cohort) and sepsis-associated organ dysfunction. Higher INR likely captures a combination of acute coagulopathy of critical illness, hepatic dysfunction, and prior anticoagulant use in elderly cardiopulmonary patients. We did not develop a formal multivariable risk score because the events-per-variable ratio was insufficient to support such a model without overfitting; instead, we report these signals as candidate variables for future, adequately powered prognostic-model development in this population.

The discrepancy between our findings (and those of Upchurch et al.) and studies in more homogeneous populations likely reflects several important physiological and methodological factors: first, ED patients requiring emergent intubation represent a fundamentally heterogeneous population with diverse underlying pathophysiology. In our cohort, patients were intubated for acute respiratory failure of various etiologies—likely including pneumonia, COPD/asthma exacerbations, pulmonary edema, aspiration, and possibly mixed shock states. In pure ventilatory failure (e.g., COPD exacerbation, neuromuscular weakness), both PaCO_2_ and ETCO_2_ tend to rise proportionally, maintaining a relatively normal gradient. In conditions with increased dead space (e.g., pulmonary embolism, severe ARDS, shock states), the gradient widens as ETCO_2_ falls relative to PaCO_2_. In high cardiac output states or mild disease, the gradient may remain narrow despite critical illness. Therefore, in a heterogeneous ED population, these opposing effects may cancel each other out at the population level, obscuring any prognostic signal from ΔCO_2_. In contrast, studies in trauma or ARDS examine more pathophysiologically uniform cohorts where increased dead space is a consistent feature.

Second, the timing of measurement is another significant difference across studies in the literature. Our measurements were obtained within the first 90 min post-intubation, capturing the immediate peri-intubation period. This timing differs from many ICU studies that examined established mechanically ventilated patients hours to days after intubation. The early post-intubation period is characterized by hemodynamic instability from sedation, positive pressure ventilation, underlying critical illness, incomplete ventilator optimization, evolving gas exchange, and transient physiological perturbations that may not reflect steady-state cardiopulmonary function. Upchurch et al. similarly measured CO_2_ parameters in the early post-intubation window and found no association with mortality. It is plausible that ΔCO_2_ requires a more stable physiological state to serve as a reliable prognostic marker and that measurements obtained after initial stabilization (e.g., 6–24 h post-intubation) might yield different results [21].

Two additional features of our cohort merit emphasis. First, the case mix is heavily weighted toward COPD exacerbation (40%) and pneumonia (64%, with substantial overlap), both of which are typically associated with elevated baseline PaCO_2_ through increased physiologic dead space and impaired CO_2_ clearance; a 90 min window post-intubation is therefore expected to remain on the upper edge of normal PaCO_2_ rather than normalizing fully. Second, our institutional standard is lung-protective ventilation with low tidal volumes (6–8 mL/kg predicted body weight), and aggressive normalization of PaCO_2_ is intentionally avoided in chronically hypercapnic patients. Together these factors explain why median PaCO_2_ stayed comparatively high throughout the observation window and reinforce the need to interpret CO_2_ trajectories in the early post-intubation period within their physiologic and ventilator-strategy context.

Third, a particularly intriguing finding in our study is that both PaCO_2_ and ETCO_2_ were significantly lower in non-survivors. To avoid a possible misinterpretation of these findings, we have clarified that the lower ETCO_2_ and PaCO_2_ observed in non-survivors should not be read as evidence that hypoventilation is protective. Several considerations argue against such a causal interpretation. First, ETCO_2_ is a well-validated marker of cardiac output and tissue perfusion in cardiac-arrest and shock states; lower ETCO_2_ in non-survivors is therefore mechanistically consistent with greater hemodynamic compromise and increased physiologic dead space rather than with a beneficial ventilatory strategy. Second, large mechanically ventilated cohorts have described a U-shaped relationship between PaCO_2_ and mortality, with the lowest survival at both extremes [27]; in COVID-19 mechanical ventilation, ventilator-induced low PaCO_2_ has been independently associated with higher mortality [28]. Third, in our cohort, the parallel decline of ETCO_2_ and PaCO_2_ in non-survivors coexisted with a significantly lower post-intubation MAP, which is more consistent with worsening perfusion than with deliberately deeper ventilation. Our findings are therefore best interpreted as confirming the role of low ETCO_2_ as an early warning sign of poor perfusion and dead-space pathology, while also underscoring the limited prognostic value of the static CO_2_ gap in heterogeneous ED-intubation populations. This pattern, while maintaining a similar ΔCO_2_, suggests that the absolute level of CO_2_—rather than the gradient—carries prognostic information. The key insight is that when both values fall in parallel, ΔCO_2_ remains unchanged despite profound underlying pathophysiology. This suggests that, in heterogeneous ED populations, ΔCO_2_ may be an insufficient metric to capture the complex interplay among ventilation, perfusion, and metabolism that determines outcome. Last, it is important to consider that survivors and non-survivors may have received different ventilator management strategies based on clinical severity. If clinicians titrated ventilation more aggressively in patients perceived as more critically ill (who subsequently had higher mortality), this could explain the observed lower PaCO_2_ and ETCO_2_ in non-survivors. However, this would reflect appropriate clinical decision-making based on illness severity rather than undermining the prognostic value of these measurements.

Our findings suggest that absolute values of ETCO_2_ and PaCO_2_ may be more clinically useful than their difference in ED intubation patients. These findings align with the broader critical care literature, which shows that ETCO_2_ is a powerful marker of cardiac output and tissue perfusion. The established use of ETCO_2_ in cardiac arrest and resuscitation as a marker of low cardiac output/ineffective compressions lends physiological plausibility to our pattern [1,2,12]. In cardiac arrest, for example, ETCO_2_ < 10 mmHg during CPR is associated with extremely poor outcomes [12], and Kolar et al. demonstrated that PetCO_2_ > 1.9 kPa (~14.3 mmHg) at 20 min of resuscitation achieved near-perfect discrimination for return of spontaneous circulation [29]. Similarly, prehospital ETCO_2_ has been shown to predict in-hospital mortality with an AUC of 0.76, likely reflecting underlying perfusion status [30]. This pattern also aligns with previous intensive care literature, which describes ETCO_2_ as a sensitive marker of perfusion adequacy and prognosis [1,2,20], whereas ΔCO_2_ may fail to provide additional early prognostic discrimination in heterogeneous critical care cohorts [13,14].

Finally, PaCO2’s intermittent differences—versus ETCO2’s prompt and sustained signal—suggest that PaCO_2_ responds more slowly and to a broader set of determinants (minute ventilation, buffering), whereas ETCO_2_ rapidly integrates perfusion–ventilation changes. This dissociation supports privileging ETCO_2_ as an “early warning” indicator in decision pathways [1,2,12].

Recent evidence points to high-sensitivity troponin T and NT-proBNP as integrators of myocardial injury and ventricular overload that independently predict mortality in critically ill patients, even in the absence of an acute coronary syndrome. Elevated NT-proBNP and troponin levels have been shown to predict mortality in ARDS, in community-acquired pneumonia, in acute exacerbations of COPD, and in undifferentiated acute dyspnea presenting to the ED. Although our study was not designed to capture these biomarkers systematically, the lower post-intubation mean arterial pressure observed in non-survivors (MAP_0_ 74.8 vs. 94.7 mmHg, *p* = 0.038) is consistent with greater hemodynamic vulnerability in this group, and the parallel decline of ETCO_2_ and PaCO_2_ that we report is mechanistically congruent with reduced cardiac output and increased physiologic dead space. We therefore consider the prospective co-measurement of hs-cTnT, NT-proBNP, and capnographic CO_2_ metrics at fixed peri-intubation time points to be a high-priority direction for future studies aiming to refine bedside risk stratification in heterogeneous ED-intubation populations.

### Limitations

This is a single-center cohort, with an effective sample size varying by time point. Therefore, we employed the last observation carried forward method to handle missing data, and no significant differences were observed between the two conditions. Second, despite adherence to capnography standards, practical issues (secretion burden, circuit leakage, T-piece/filter changes) could bias ETCO_2_ downward. Micro-adjustments in ventilator settings (FiO_2_, tidal volume, PEEP, minute ventilation) and depth of sedation/analgesia were not fully modeled, potentially leaving residual confounding in the PaCO_2_–ETCO_2_ relationship.

Third, the etiology of acute respiratory failure in our cohort was deliberately heterogeneous, reflecting real-world ED practice. This heterogeneity affects baseline CO_2_ physiology in important and divergent ways: COPD patients frequently present with chronically elevated baseline PaCO_2_ (compensated respiratory acidosis) and reduced minute-ventilation reserve, pulmonary embolism patients show widened CO_2_ gaps from increased dead space, ARDS and shock states are dominated by low cardiac output and low ETCO_2_, and patients with pure ventilatory failure may show parallel rises in PaCO_2_ and ETCO_2_ with a preserved gradient. These competing physiologies likely cancel out at the population level when one examines a static CO_2_ gap, which may help explain why ΔCO_2_ failed to discriminate survivors from non-survivors in our cohort and in the comparable Upchurch et al. study. Studies in pathophysiologically homogeneous populations (e.g., trauma, post-cardiac arrest, suspected sepsis) avoid this dilution effect, which is consistent with the larger CO_2_-gap signal reported in those settings.

Fourth, because contributing diagnoses overlapped substantially (29% of patients had two or more), single-diagnosis subgroup analyses are descriptive and cannot support causal inference about the contribution of any individual disease to mortality or to CO_2_ trajectories. Adequately powered, pathophysiologically homogeneous cohorts are needed to dissect the specific contribution of each underlying disease.

Fifth, cardiac biomarkers such as high-sensitivity troponin T and NT-proBNP were not pre-specified in the protocol and were obtained only when clinically indicated; their values are therefore missing-not-at-random, and we did not perform formal correlation analysis between these biomarkers and ΔCO_2_ or the ETCO_2_/PaCO_2_ ratio. Prospective co-measurement of cardiac biomarkers with capnographic CO_2_ metrics is an important next step. Finally, mortality was assessed at 1 and 3 months, which may be too long for evaluating an acute predictor.

## 5. Conclusions

In the early post-intubation window, low ETCO_2_ values were consistently associated with both 1- and 3-month mortality, whereas PaCO_2_ provided intermittent incremental information, and ΔCO_2_ (PaCO_2_–ETCO_2_) did not add prognostic discrimination in this heterogeneous cohort. These findings should not be interpreted as supporting permissive or deliberate hypoventilation; rather, lower ETCO_2_ in non-survivors most likely reflects worse cardiac output, greater physiologic dead space, and greater hemodynamic vulnerability, and is best regarded as an early bedside warning sign in the peri-intubation period.

## Figures and Tables

**Table 1:** Demographics, selected baseline laboratory parameters, and contributing diagnoses were compared between survivors and non-survivors at 30 days. Contributing diagnoses are not mutually exclusive; 29 patients (29.0%) carried more than one contributing diagnosis, most commonly pneumonia superimposed on COPD (n = 18) or on chronic heart failure (n = 11). Single-diagnosis subgroup comparisons should therefore be interpreted as descriptive rather than causal.

*N* = 100	Outcome 1 Month		*p*
	Non-Survivors, (*N* = 64)	Survivors, (*N* = 36)	
Demographics			
Age, years	76.5 (69–85)	74 (63–80.3)	0.037
Female, *n* (%)	24 (37.5)	12 (33.3)	0.829
Vital signs presentation (*p*)			
MAP_p_, mmHg	93.0 (76.7 to 113.1)	95.7 (78.6 to 120.0)	0.520
HR_p_, bpm	113.5 (100.0 to 130.3)	102.0 (88.5 to 125.3)	0.130
RR_p_/min	33 (30 to 40)	35 (30 to 40)	0.709
pSO_2p_, %	80 (75 to 85)	80 (75 to 85)	0.949
Temperature_p_, C	36.5 (36.0 to 36.8)	36.7 (36.0 to 36.9)	0.582
after intubation, (0. min)			
MAP_0_, mmHg	74.8 (63.6 to 102.0)	94.7 (79.6 to 101.8)	0.038
HR_0_, bpm	110.5 (96.8 to 127.0)	98.5 (88.0 to 115.0)	0.060
Lab values at presentation			
pH	7.29 (7.16 to 7.38)	7.18 (7.12 to 7.26)	0.005
Base excess, mEq/L	−3.9 (−8.6 to 1.1)	−3.6 (−9.0 to 2.4)	0.937
Lactate, g/dL	3.2 (2.1 to 5.3)	2.7 (1.8 to 6.4)	0.709
WBC, ×1000 /mm^3^	12.9 (9.7 to 16.8)	13.5 (9.5 to 19.1)	0.755
Hgb, g/dL	12.1 (10.3 to 13.4)	12.4 (10.1 to 14.8)	0.453
Procalcitonin (N, 61:33)	0.30 (0.10 to 1.63)	0.13 (0.07 to 0.44)	0.042
CRP (N, 62:34)	68.5 (25.4 to 158.3)	17.2 (8.5 to 80.7)	0.004
INR (N, 56:30)	1.31 (1.17 to 1.43)	1.16 (1.07 to 1.30)	0.009
Contributing diagnosis			
Pneumonia	49 (76.6)	15 (41.7)	0.001
Congestive heart failure	22 (34.4)	17 (47.2)	0.286
COPD	22 (34.4)	18 (50.0)	0.142
Pulmonary embolism	8 (12.5)	0 (0.0)	0.048
Lung cancer	10 (15.6)	1 (2.8)	0.092
Restrictive lung disease	2 (3.1)	0 (0.0)	0.535

All continuous variables were reported with median (IQR). Proportions were compared by Fisher’s exact test, medians were compared by the Mann–Whitney U test. MAP: mean arterial pressure, HR: heart rate, RR: respiratory rate, MAP_0_: mean arterial pressure at the 0 min point following intubation, CRP: C-reactive protein, INR: international normalized ratio.

**Table 2:** Change in CO_2_ parameters from 0 to 90 min after intubation, compared between 30-day survival groups. The upper rows show the complete-case analysis (n = 57: 23 survivors and 34 non-survivors with paired 0 and 90 min measurements). The lower rows (‘LOCF’) re-run the same comparison on the full cohort (N = 100: 36 survivors and 64 non-survivors) using last observation carried forward imputation for missing 60 and 90 min values. The discrepancy in survivor counts between Table 1 and Table 2 reflects this complete-case constraint, not a different patient population.

	Non-Survivors, *n* = 34	Survivors, *n* = 23	Mean Difference (95% CI of the Difference)
Δ PaCO_2_ (0–90 min)	4.5 (21.0)	13.0 (23.7)	8.5 (−3.5 to 20.5)
Δ ETCO_2_ (0–90 min)	3.0 (11.2)	11.2 (17.0)	8.2 (0.7 to 15.7)
Δ ΔCO_2_ (0–90 min)	1.2 (20.0)	2.5 (24.3)	1.3 (−10.5 to 13.1)
LOCF	*n* = 64	*n* = 36	
Δ PaCO_2_ (0–90 min)	1.3 (19.3)	8.9 (22.1)	7.6 (−0.9 to 16.0)
Δ ETCO_2_ (0–90 min)	3.7 (11.9)	8.2 (16.6)	2.9 (−1.2 to 10.1)
Δ ΔCO_2_ (0–90 min)	−2.4 (17.7)	1.6 (20.0)	4.0 (−3.7 to 11.6)

CI: confidence interval, Δ: delta, PaCO_2_: partial pressure of carbon dioxide, LOCF: last observation carried forward, ETCO_2_: end-tidal carbon dioxide.

## Data Availability

The data presented in this study are available on request from the corresponding author.

## Abbreviations

ABG	Arterial blood gas
BPM	Beats per minute
COPD	Chronic obstructive pulmonary disease
CO_2_	Carbon dioxide
CRP	C-reactive protein
ΔCO_2_	Delta carbon dioxide
DBP	Diastolic blood pressure
DBP_0_	Diastolic blood pressure at the 0 min point following intubation
DBP_0_	Mean arterial pressure at the 0 min point following intubation
ETCO_2_	End-tidal carbon dioxide
ICU	Intensive care unit
INR	International normalized ratio
LOCF	Last observation carried forward
MAP	Mean arterial pressure
PaO_2_	Partial pressure of carbon dioxide
PE	Pulmonary embolism
PEEP	Positive end-expiratory pressure
RR	Respiratory rate
SBP	Systolic blood pressure
V/Q	Ventilation/perfusion
